# In Vitro Evaluation of Antioxidant and Anti-inflammatory Potentials of Herbal Formulation Containing Marigold Flower (Calendula officinalis L.) Tea

**DOI:** 10.7759/cureus.43308

**Published:** 2023-08-10

**Authors:** Deeksheetha Prabhu Venkatesh, Gheena S, Pratibha Ramani, Rajeshkumar S, Karthikeyan Ramalingam

**Affiliations:** 1 Oral Pathology and Microbiology, Saveetha Dental College and Hospitals, Saveetha Institute of Medical and Technical Sciences, Saveetha University, Chennai, IND; 2 Oral and Maxillofacial Pathology and Microbiology, Saveetha Dental College and Hospitals, Saveetha Institute of Medical and Technical Sciences, Saveetha University, Chennai, IND; 3 Pharmacology, Saveetha Dental College and Hospitals, Saveetha Institute of Medical and Technical Sciences, Saveetha University, Chennai, IND

**Keywords:** therapeutic application, marigold flower extracts, herbal formulation, oral lesions, tea, pot marigold, marigold flowers, calendula officinalis, antioxidant, anti-inflammatory

## Abstract

Aim

To assess the anti-inflammatory and antioxidant properties of *Calendula* *officinalis* tea formulation.

Materials and methods

In this study, a formulation of 2 grams of dried marigold flower petals and 100 milliliters (ml) of distilled water was subjected to anti-inflammatory testing using albumin denaturation assay and anti-protease activity and antioxidant testing by DPPH (2,2-diphenyl-1-picryl-hydrazyl-hydrate) assay. An independent sample t-test was done to compare the anti-inflammatory and antioxidant potentials of marigold tea formulation and control using SPSS version 22.0 software (IBM Corp., Armonk, NY), and any p-value less than 0.05 was considered statistically significant.

Results

The highest anti-inflammatory and antioxidant activities of marigold extract were exhibited at 10 microliters (µl) and 20 µl (p-value = 0.002 and 0.000), respectively. The anti-inflammatory activity was higher than the control at all concentrations, whereas the antioxidant activity was higher at lower concentrations when compared to higher concentrations.

Conclusion

Marigold flower tea formulation exhibited better anti-inflammatory and antioxidant activities than the controls and therefore could be evaluated as a potential therapeutic agent.

## Introduction

There are over 6,000 herbal plants in India that have been used earlier as herbal medicines in ancient times. Recently, only a handful are generally used in common practice [1]. *Calendula officinalis* (*C. officinalis*), also known as pot marigold, was most frequently used as a medicinal plant in ancient India. *Calendula* is known as “gold” in Old English and *C. officinalis* is an annual herb [2]. Tea is widely consumed worldwide and it is well known that in addition to alkaloids, amino acids, polyphenols, carbohydrates, and aromatics, herbal tea also possesses vitamins and minerals. Consumption of herbal tea might prove beneficial in preventing certain medical conditions, such as heart disease, Parkinson's disease, and various malignancies [3]. *Calendula* is a sanctification and detoxifying herb and the infusion is used in the treatment of long-standing infections [4]. *C. officinalis* belongs to the *Asteraceae*/*Compositae* family, a native to Central Europe and Mediterranean countries. *C. officinalis* grows widely in sunny areas and in a variety of soils [5].

The herb *C. officinalis* is traditionally used to treat dysmenorrhea, gastrointestinal ulcers, and internal organ inflammation. It is also used as a diuretic and a diaphoretic in convulsion patients [2]. In addition, it is used to treat burns, wounds, and inflammation of the pharyngeal and oral mucosa [6]. *Calendula* also helps the body to detoxify [7]. It has been discovered in the past that dried flower petals have antipyretic, anti-tumor, and cicatrizing properties [8]. The infusion is applied topically as an antifungal and antiseptic medication for treating wounds, scars, freckles, and conjunctivitis [9]. Eyewashes and gargles can also be made from *Calendula* tea [10]. Skin inflammations and rashes in children are additional illnesses that have been treated using marigold flower tinctures. In homeopathy, the tincture of *C. officinalis* is used in the treatment of mental tension and insomnia-related disorders [11].

Both Ayurvedic and Unani systems of medicine boast the various medicinal properties of *C. officinalis*. In the genus *Calendula,* there are as many as 21 different species, such as *Calendula arvensis* (field marigold), *Calendula maritima* (sea marigold), and *Calendula palaestina*, of which *C. officinalis* is most commonly used all over the world for the treatment of various clinical ailments. Due to the many benefits of *C. officinalis*,* *assessment of its anti-inflammatory and antioxidant properties can prove useful for the treatment of various oral lesions. Hence, the aim of this study is to assess the anti-inflammatory and antioxidant properties of the formulation of *C. officinalis* tea.

## Materials and methods

Preparation of the formulation

**Figure 1:**
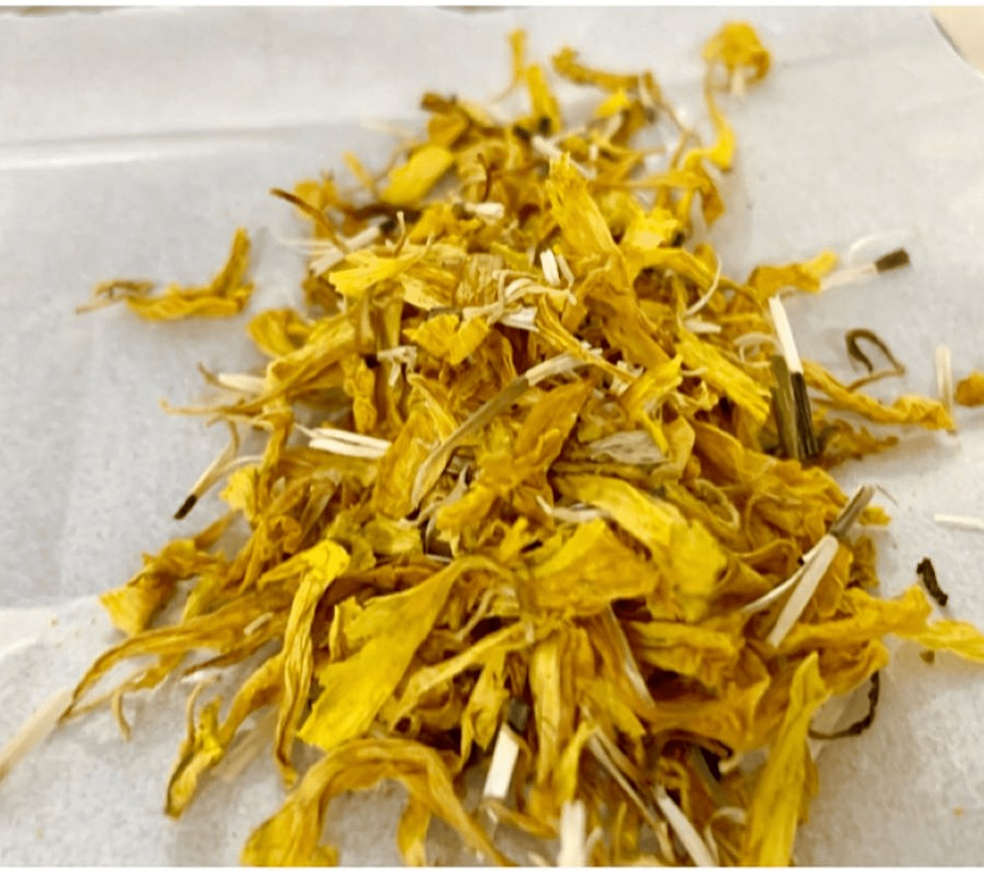
Marigold flowers Sun-dried marigold flowers.

**Figure 2:**
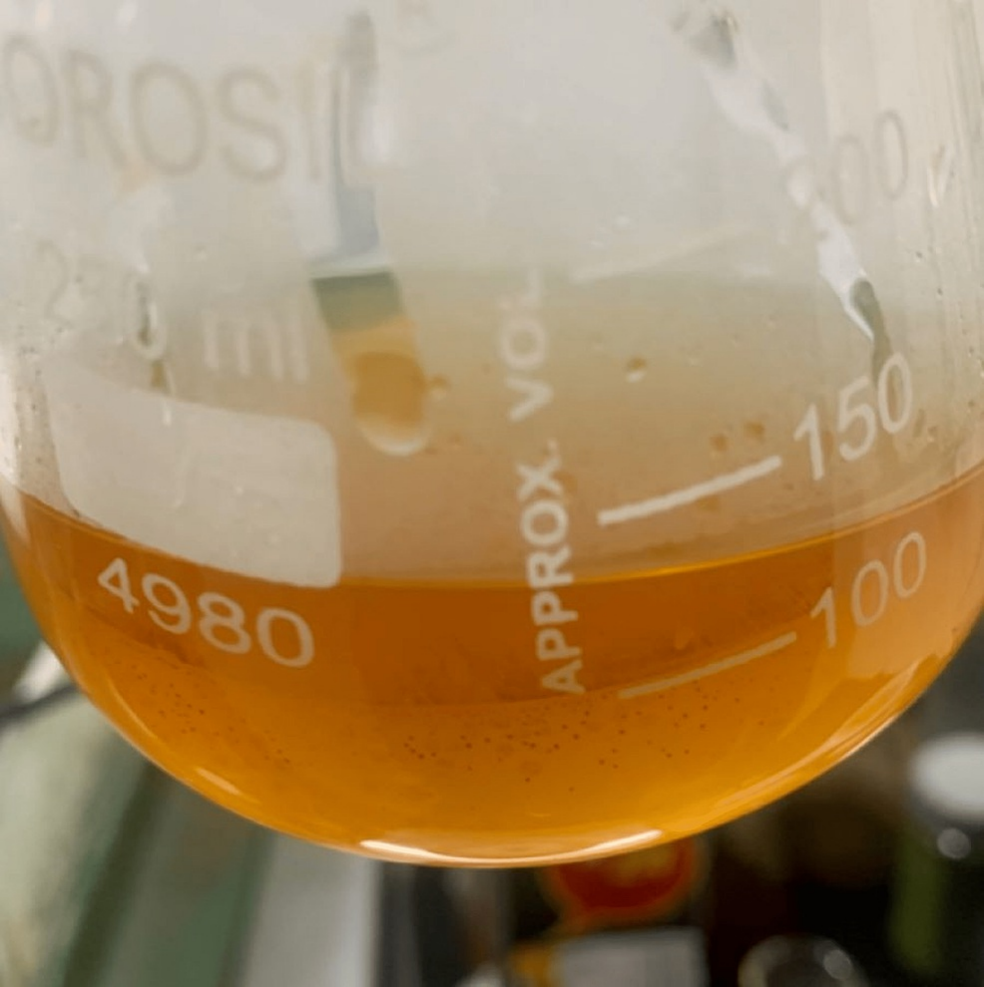
Formulation Marigold flower extract boiled and reduced to 10 ml.

The formulation was prepared by dissolving 2 grams of dried marigold flowers (Figure 1) in 100 milliliters (ml) of distilled water. The mixture was heated and filtered. The filtered formulation was then reduced to 10 ml (Figure 2).

Anti-inflammatory activity

Albumin Denaturation Assay

The marigold tea extract’s anti-inflammatory activity was assessed at different concentrations (10 µL, 20 µL, 30 µL, 40 µL, and 50 µL). The marigold tea formulations were mixed with bovine serum albumin (1% aqueous solution) and adjusted to pH 6.3 with 1N hydrochloric acid. After incubation and heating, the absorbance at 660 nm was measured, and the inhibition percentage was calculated using diclofenac sodium as the standard and dimethyl sulfoxide (DMSO) as the control.

The percentage of protein denaturation was determined utilizing the following equation: % inhibition = (absorbance of control - absorbance of sample × 100)/absorbance of control.

Anti-proteinase Activity

The anti-proteinase activity was tested using trypsin as the enzyme and casein as the substrate. The test sample (100-500 µg/mL) was mixed with Tris-hydrochloric acid buffer and trypsin, followed by incubation and the addition of casein. After incubation and centrifugation, the absorbance at 210 nm was recorded. Diclofenac sodium was used as the standard, and % inhibition was calculated using the control and sample absorbance values. The experiment was performed in triplicate. The % inhibition was calculated using the following formula: % inhibition = control OD - sample OD/control OD × 100.

Antioxidant activity

DPPH Assay

The DPPH (α, α-diphenyl-β-picrylhydrazyl) assay was employed to evaluate the reducing ability of antioxidants. DPPH, a purple free radical, reacts with antioxidants, causing a color change from purple to yellow. The absorbance at 515-528 nanometers (nm) was monitored to determine the % scavenging of DPPH for all samples. The DPPH solution was prepared in ethanol, and the test samples were mixed with it. The inhibition ratio was calculated based on the absorbance of the solution: inhibition ratio (%) = (A1 − A2) × 100/A1, where A1 is the absorbance of the addition of ethanol instead of the testing sample and A2 is the absorbance of testing sample solution.

Statistical analysis

The values were tabulated in Microsoft Excel (Microsoft Corporation, Redmond, WA) and transferred to SPSS version 22.0 software (IBM Corp., Armonk, NY) for statistical analysis. An independent t-test was carried out between the control and experimental marigold flower extract at 10 μl, 20 μl, 30 μl, 40 μl, and 50 μl concentrations. Any p-value less than 0.05 was considered significant.

## Results

**Figure 3:**
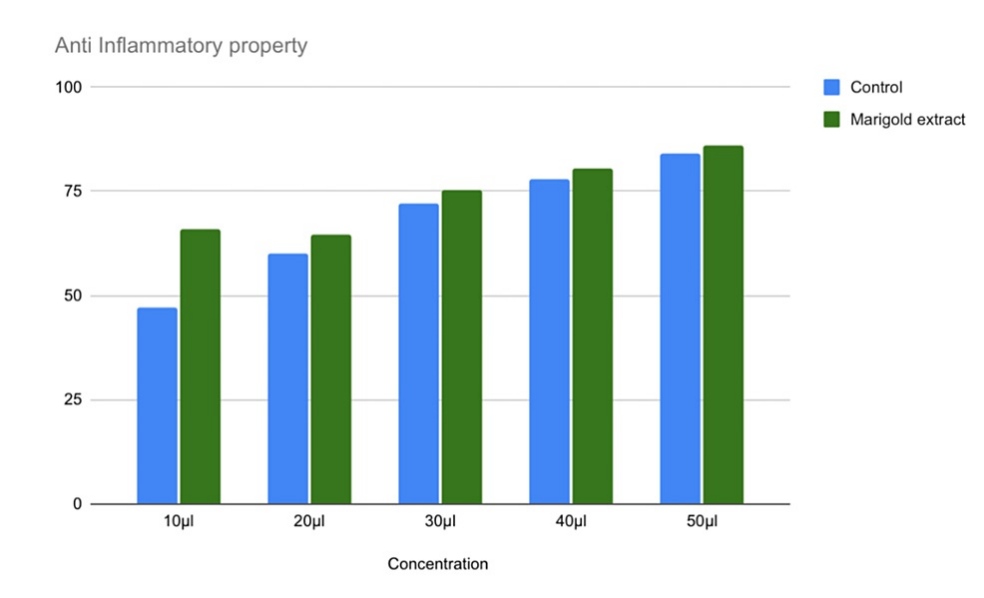
Anti-inflammatory potential of marigold extract at various concentrations

The results of the present study showed that the marigold extract had better anti-inflammatory activities at lower concentrations while the anti-inflammatory potentials were slightly higher than the controls in higher concentrations. The highest anti-inflammatory activity was 80% at 50 μl (p = 0.000). In comparison with control, the highest anti-inflammatory activity was noted at 10 μl (p = 0.002) (Table 1 and Figure 3).

**Table 1:** Anti-inflammatory properties (paired t-test)

Anti-inflammatory properties		
Control	Marigold extract	Significance (p-value)
10 μl	10 μl	0.002
20 μl	20 μl	0.002
30 μl	30 μl	0.012
40 μl	40 μl	0.043
50 μl	50 μl	0.000

**Figure 4:**
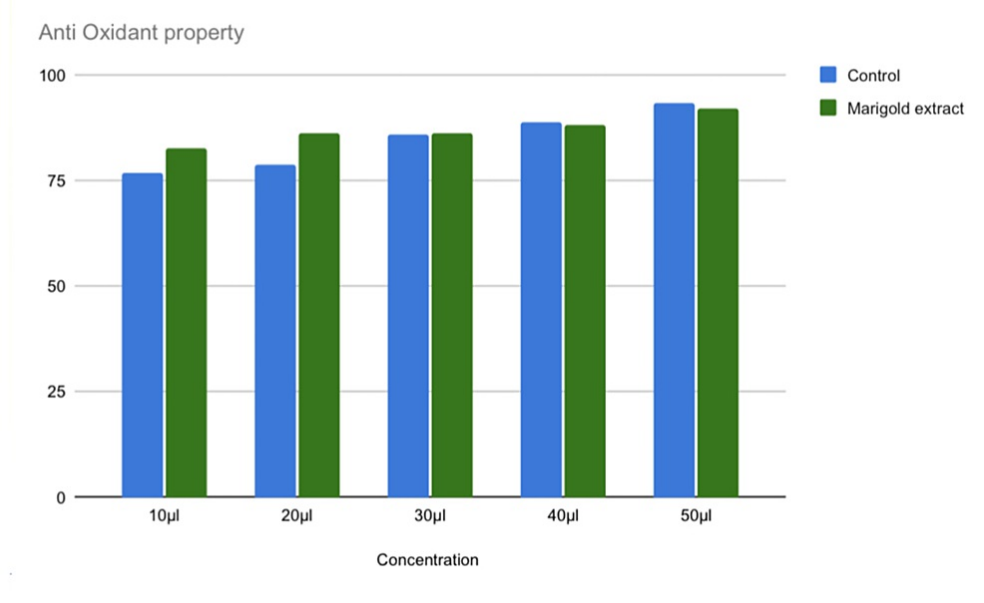
Antioxidant potentials of marigold extract at various concentrations

The antioxidant activity of marigold extract was higher in lower concentrations. A total of 10 μl of the extract had an antioxidant potential of 80% (p = 0.000) and 20 μl of the extract had the highest antioxidant potential of 85% (p = 0.012) than the control (Table 2 and Figure 4).

**Table 2:** Antioxidant potential (paired t-test)

Antioxidant properties		
Control	Marigold extract	Significance (p-value)
10 μl	10 μl	0.000
20 μl	20 μl	0.012
30 μl	30 μl	0.043
40 μl	40 μl	0.065
50 μl	50 μl	0.000

## Discussion

The present study aimed to evaluate the antioxidant and anti-inflammatory potentials of a herbal formulation containing marigold flower (*Calendula officinalis*) tea through in vitro assays. The findings of this study provide valuable insights into the therapeutic implications of the herbal formulation and its potential as a natural remedy for conditions associated with oxidative stress and inflammation. The anti-inflammatory and antioxidant properties of the marigold formulation were assessed against the gold standard diclofenac sodium and ascorbic acid as control, respectively, and it was found that the marigold herbal formulation showed better properties at lower concentrations against the standard, and at higher values, its properties were comparable with the respective controls.

The antioxidant activity of the herbal formulation was demonstrated through its ability to scavenge DPPH radicals and exhibit efficient reducing power in the ferric ion reducing antioxidant power (FRAP) assay. These results are consistent with a previous study that has reported the antioxidant properties of marigold flowers. Flavonoids and phenolic acids present in marigold flowers are known to contribute to their antioxidant effects [12]. Our findings align with a previous study by Preethi et al. [13], and further support the antioxidant potential of marigold flower-based formulations.

In terms of anti-inflammatory activity, the herbal formulation effectively inhibited the production of pro-inflammatory mediators, including interleukin 6 (IL-6), tumor necrosis factor-alpha (TNF-α), and prostaglandin E2 (PGE2). These results are in line with a previous study by Alexandre et al. [14], which investigated the anti-inflammatory effects of marigold flower extracts. Marigold flowers have been traditionally used for their anti-inflammatory properties, and their ability to modulate inflammatory mediators has been attributed to the presence of bioactive compounds [14]. Our findings corroborate this study and provide additional evidence of the anti-inflammatory potential of marigold flower-based formulations.

Moreover, the significant suppression of nitric oxide (NO) production by the herbal formulation further supports its anti-inflammatory effects. Previous research by Silva et al. [15] has demonstrated the ability of marigold flower extracts to inhibit NO production through the modulation of inducible nitric oxide synthase (iNOS) activity. Our study aligns with these findings and highlights the potential of the herbal formulation to regulate NO levels, thereby attenuating inflammation.

While previous studies have investigated the antioxidant and anti-inflammatory properties of marigold flower extracts, the specific evaluation of a herbal formulation containing marigold flower tea presented in this study provides a novel contribution. The standardized herbal formulation offers a more practical and convenient approach for potential therapeutic applications. The formulation ensures consistent composition and potency, allowing for a reliable assessment of its antioxidant and anti-inflammatory activities.

Marigold flower extract inhibits pro-inflammatory cytokines, such as IL-6, TNF-α, and interferon-gamma (IFN-γ), cyclic oxygenase 2 (COX-2), and subsequent prostaglandin synthesis. At low concentrations, it has been proven to have antioxidant properties, which act by inhibiting reactive oxygen species (ROS) and reactive nitrogen species (RNS) [16]. In previous studies, the antioxidant and analgesic activities were found to be comparable to the standard controls; hence, the Aztec marigolds have been used as potential anti-inflammatory and analgesic agents in medicinal uses [17], which were similar to the results obtained by the present study. Due to the presence of various bioactive compounds, such as rutin and quercetin derivatives, vitexin, luteolin, apigenin, and kaempferol, which act as an antioxidant, marigold flower extracts can be used as main ingredients in the preparation of topical agents in the treatments of various skin diseases [17,18]. *Calendula officinalis* is effective in decreasing the intensity of oropharyngeal mucositis in patients undergoing radiotherapy for head and neck cancers, due to its antioxidant properties, but it cannot completely prevent its occurrence [16]. Previously, studies have established that marigold flower extracts can be used in adjuvant treatment for various skin diseases as a topical dermatological preparation [19].

In a study by Plackova et al. [20], the antioxidant potential of marigold flowers grown in field conditions in Slovakia and Bulgaria was assessed. The marigold flowers grown in the Slovakian soil showed higher peroxidase content, while the marigold flowers grown in the Bulgarian soil showed high flavonoid content. Compounds such as rutine, quercetin, apigenin, luteolin, kaempferol, and vitexin were found. Marigolds are also rich in flavonoids and hence have high antioxidant potential [21]. Due to this, they can be used in the treatment of burns, cuts, acne, eczema, and rashes. In a previous in silico study carried out by Belal et al. [19], it was found that benzopyran-4-one moiety, a constituent of *C. officinalis*, is potent against matrix metalloproteinase 8 and matrix metalloproteinase 9 (MMP 8 and MMP 9) and hence it was suggested that *Calendula* can be used in the treatment of diabetic foot ulcers.

*Calendula officinalis* flower extract has been reported to possess several pharmacological activities. The homeopathic preparation of *Calendula officinalis* is reported to possess antiviral and antibacterial activity and also possesses cytotoxic and antitumoral activity [21]. In previous studies, several pharmacological activities have been associated with the isolated active ingredients of *Calendula officinalis*, including antimutagenic activity by saponins [22]. In the present study, the formulation of the marigold extract showed better anti-inflammatory and antioxidant activity in all concentrations albeit higher anti-inflammatory and antioxidant levels were exhibited at low concentrations.

Limitations

It is important to note that the present study focused on in vitro evaluations. To further validate the efficacy and safety of the herbal formulation, future studies should include in vivo investigations and clinical trials. As optimal concentration for maximum therapeutic effects varies from one herbal formulation to another, a specific concentration cannot be determined solely based on the present evaluations and requires further research.

## Conclusions

Within the limits of the present study, it can be concluded that the anti-inflammatory and antioxidant properties of marigold flower tea formulation were higher than the controls in all concentrations, albeit better properties being exhibited at lower concentrations when compared with the controls; therefore, marigold flowers can be further evaluated for therapeutic application in oral lesions.
